# Corrigendum to ‘p53 balances between tissue hierarchy and anarchy’

**DOI:** 10.1093/jmcb/mjz111

**Published:** 2019-12-26

**Authors:** Gabriela Koifman, Ronit Aloni-Grinstein, Varda Rotter

**Affiliations:** 1 Department of Molecular Cell Biology, the Weizmann Institute of Science, Rehovot 7610001, Israel; 2 Department of Biochemistry and Molecular Genetics, Israel Institute for Biological Research, Ness Ziona, Israel

In this article (p.559, third paragraph), the reference in ‘The identified ESC gene signature-derived genes correlated with poor patient survival and human tumors harboring p53 hotspot mutations (Lonetto et al., 2018)’ was incorrectly cited. It should be changed to ‘(Koifman et al., 2018)’. The conclusions of the review are not affected and the authors apologize for this error.

The corrected text is shown below:

Recently, we have established an *in vitro* MSC p53-based system, which permitted the tracing of a cancer multistep process of ASCs and their conversion into CSCs. Eventually, we established aggressive mutant p53-expressing CSC-like cell lines that allowed the identification of a gene signature entailing embryonic specific genes in conjunction with cancer-associated genes. The identified ESC gene signature-derived genes correlated with poor patient survival and human tumors harboring p53 hotspot mutations (Koifman et al., 2018). Interestingly, a recent study from our laboratory showed that the established CSC-like cell lines exhibited a mutant-dependent metabolic profile that included the increment of mitochondrial mass and oxidative metabolism (Lonetto et al., 2018). Moreover, mutant p53 was shown to upregulate the mevalonate pathway genes that were shown to be important for the self-renewal and survival of breast CSCs (Ginestier et al., 2012).

The below reference should be added to the **References** section:
